# Hypokalemic periodic paralysis: a 3-year follow-up study

**DOI:** 10.1007/s00415-023-11964-z

**Published:** 2023-09-01

**Authors:** Sonja Holm-Yildiz, Thomas Krag, Nanna Witting, Britt Stævnsbo Pedersen, Tina Dysgaard, Louise Sloth, Jonas Pedersen, Rebecca Kjær, Linda Kannuberg, Julia Dahlqvist, Josefine de Stricker Borch, Tuva Solheim, Freja Fornander, Anne-Sofie Eisum, John Vissing

**Affiliations:** Copenhagen Neuromuscular Center, Department of Neurology 8077, Rigshospitalet, University of Copenhagen, Inge Lehmanns Vej 8, 2100 Copenhagen, Denmark

**Keywords:** Hypokalemic periodic paralysis, Whole-body muscle MRI, Myopathy, Permanent muscle weakness

## Abstract

**Background and objectives:**

Primary hypokalemic periodic paralysis (HypoPP) is an inherited channelopathy most commonly caused by mutations in *CACNA1S*. HypoPP can present with different phenotypes: periodic paralysis (PP), permanent muscle weakness (PW), and mixed weakness (MW) with both periodic and permanent weakness. Little is known about the natural history of HypoPP.

**Methods:**

In this 3-year follow-up study, we used the MRC scale for manual muscle strength testing and whole-body muscle MRI (Mercuri score) to assess disease progression in individuals with HypoPP-causing mutations in *CACNA1S.*

**Results:**

We included 25 men (mean age 43 years, range 18–76 years) and 12 women (mean age 42 years, range 18–76 years). Two participants were asymptomatic, 21 had PP, 12 MW, and two PW. The median number of months between baseline and follow-up was 42 (range 26–52). Muscle strength declined in 11 patients during follow-up. Four of the patients with a decline in muscle strength had no attacks of paralysis during follow-up, and two of these patients had never had attacks of paralysis. Fat replacement of muscles increased in 27 patients during follow-up. Eight of the patients with increased fat replacement had no attacks of paralysis during follow-up, and two of these patients had never had attacks of paralysis.

**Discussion:**

The study demonstrates that HypoPP can be a progressive myopathy in both patients with and without attacks of paralysis.

## Introduction

Primary hypokalemic periodic paralysis (HypoPP) is a rare autosomal dominantly inherited skeletal muscle channelopathy with an estimated prevalence of 1–1.5:100,000 [1, 2]. HypoPP is most commonly caused by mutations in the calcium channel gene, *CACNA1S* [1, 3–7]. The most frequent pathogenic variants are p.R528H and p.R1239H, accounting for up to 70–80% of cases caused by mutations in *CACNA1S* [8].

HypoPP, caused by mutations in *CACNA1S,* can present with different phenotypes. Most patients present in adolescence with periodic paralysis (PP) lasting from a couple of hours to days [9] and some patients present with a phenotype of proximal weakness without periodic paralysis (PW) [2, 9]. Furthermore, some patients with a phenotype of PP develop permanent weakness with time, a phenotype of mixed weakness (MW) [2, 9–11]. No large follow-up studies of HypoPP have been published, so little is known of the natural history of HypoPP. In a previous cross-sectional study, we have shown that permanent muscle weakness is accompanied by fat replacement on muscle MRI and that patients with normal muscle strength also can have abnormal fat replacement on muscle MRI. Furthermore, we showed that both muscle weakness and fat replacement on muscle MRI are associated with age [2]. This indicates that HypoPP in some patients can cause a progressive myopathy and that MRI reveals signs of myopathy earlier than clinical evaluation.

In this prospective, follow-up study, we aimed to describe the natural history of HypoPP caused by pathogenic variants in *CACNA1S.*

## Methods

### Study design

This observational follow-up study was conducted between March 15, 2017, and February 15, 2022, at the Neuromuscular Center Rigshospitalet, Copenhagen.

### Participants

Inclusion criteria were adults (≥ 18 years) with a confirmed HypoPP-causing mutation in CACNA1S. Exclusion criteria were contraindications for MRI or comorbidity that would confound the interpretation of strength and MRI. All participants have participated in a larger cross-sectional study, and baseline data from that study were used in the present study [2].

### Clinical evaluation

Muscle strength (shoulder abduction and adduction, elbow, wrist, finger, hip, knee, ankle flexion, and extension, as well as hip abduction) was tested using the Medical Research Council (MRC) scale for manual muscle strength testing. Trunk flexion was assessed in the supine position and graded (grade 0: the patient cannot tilt the pelvis posteriorly to flex the lumbar spine, no muscle contractions are palpable; grade 1: the patient cannot tilt the pelvis posteriorly to flex the lumbar spine muscle, but contractions are palpable; grade 2: the patient lifts the head and scapula off the plinth, with the arms by the side; grade 3: the patient lifts the head and scapula off the plinth, with the arms held in front of the trunk; grade 4: the patient lifts the head and scapula off the plinth, with the arms positioned across the chest; grade 5: the patient lifts the head and scapula off the plinth, with the hands beside the ears). Back extension strength was assessed in the prone lying position and graded (grade 0: the xiphoid process cannot be lifted, and no muscle contractions are visible or palpable; grade 1: the xiphoid process cannot be lifted but muscle contractions are visible or palpable; grade 2: the xiphoid process is just lifted with arms by the sides; grade 3: the patient can extend part of the full movement; grade 4: full movement with hands behind the lower back; grade 5: full movement with hands behind the head) [13]. A decline in muscle strength was defined as a one-point decrease on the MRC scale from baseline to follow-up in at least one of the assessed muscle groups. All patients were assessed by the same investigator at both baseline and follow-up.

### MRI

Whole-body muscle MRI, 3.0T scanner was performed. The protocol included axial T1-weighted imaging (slide thickness of 6 mm). Fat replacement of muscles (deltoid, supraspinatus, infraspinatus, multifidus, erector spinae, psoas, iliacus, gluteus medius, gluteus maximus, the muscles of the thigh and the gastrocnemius medius and lateralis, soleus, tibialis anterior and posterior in the calves) was quantified by applying the Mercuri score (score 1 normal appearance; score 2 mild involvement comprising less than 30% of the volume of the muscle; score 3 moderate involvement comprising 30–60% of the volume of the muscle; score 4 severe involvement with a fuzzy appearance due to confluent areas of increased signal, or an end-stage appearance, with muscle replaced by increased density connective tissue and fat) [14]. The muscles were evaluated bilaterally. An increase in fat replacement of muscles was defined as a one-point increase in the Mercuri score from baseline to follow-up in at least one of the assessed muscles. All scans were reviewed by the same investigator at baseline and follow-up.

### Statistical analyses

Values are mean ± SD unless otherwise stated. Differences in mean were calculated using an independent t-test. Changes in muscle strength and fat replacement of muscle MRI were described by expressing number of patients with changes.

## Results

### Participants

We invited 54 participants from the previously published cross-sectional study with 55 participants to a follow-up visit (one participant was deceased during the follow-up). Seventeen participants were not interested in participating in a follow-up visit. The participants that declined to participate in follow-up represented all phenotypes; two were AS, nine had PP, five had MW, and one had PW. We included the 37 individuals that agreed to participate. The participants were from 18 unrelated families with HypoPP-causing mutations in *CACNA1S.* Twenty-five men (mean age 46 years, range 18–76 years) and 12 women (mean age 42 years, range 18–76 years). All except one, who was heterozygous for the mutation p.R1239H (NM_000069.2:c.3716G > A), were heterozygous for the p.R528H mutation (NM_000069.2:c.1583G > A. Two participants were asymptomatic, 21 had PP, 12 MW, and two PW. Medication was very variable (Table 1). Median months between baseline and follow-up was 42 (range 26–52).

**Table 1:** Phenotype and medication

	Decline in muscle strength *n = *11	Increase in fat replacement on muscle MRI *n = *27	Stable muscle strength and unchanged muscle MRI *n = *10
Phenotype			
Asymptomatic	0	0	2
Periodic paralysis	0	14	7
Mixed weakness	9	11	1
Permanent weakness	2	2	0
Medication			
Potassium, periodic	7	17	4
Potassium, constant	6	28	4
Triamterene	0	1	0
Acetalazomid	2	2	1
No medication	3	1	3
Mercuri at baseline			
1	0	2	7
2	3	12	2
3	1	4	1
4	7	9	0

Medication during follow-up. None of the participants were treated with diclofenamide or spironolactone. Mercuri score is from the most severely affected muscle at baseline

### Muscle strength

**Fig. 1:**
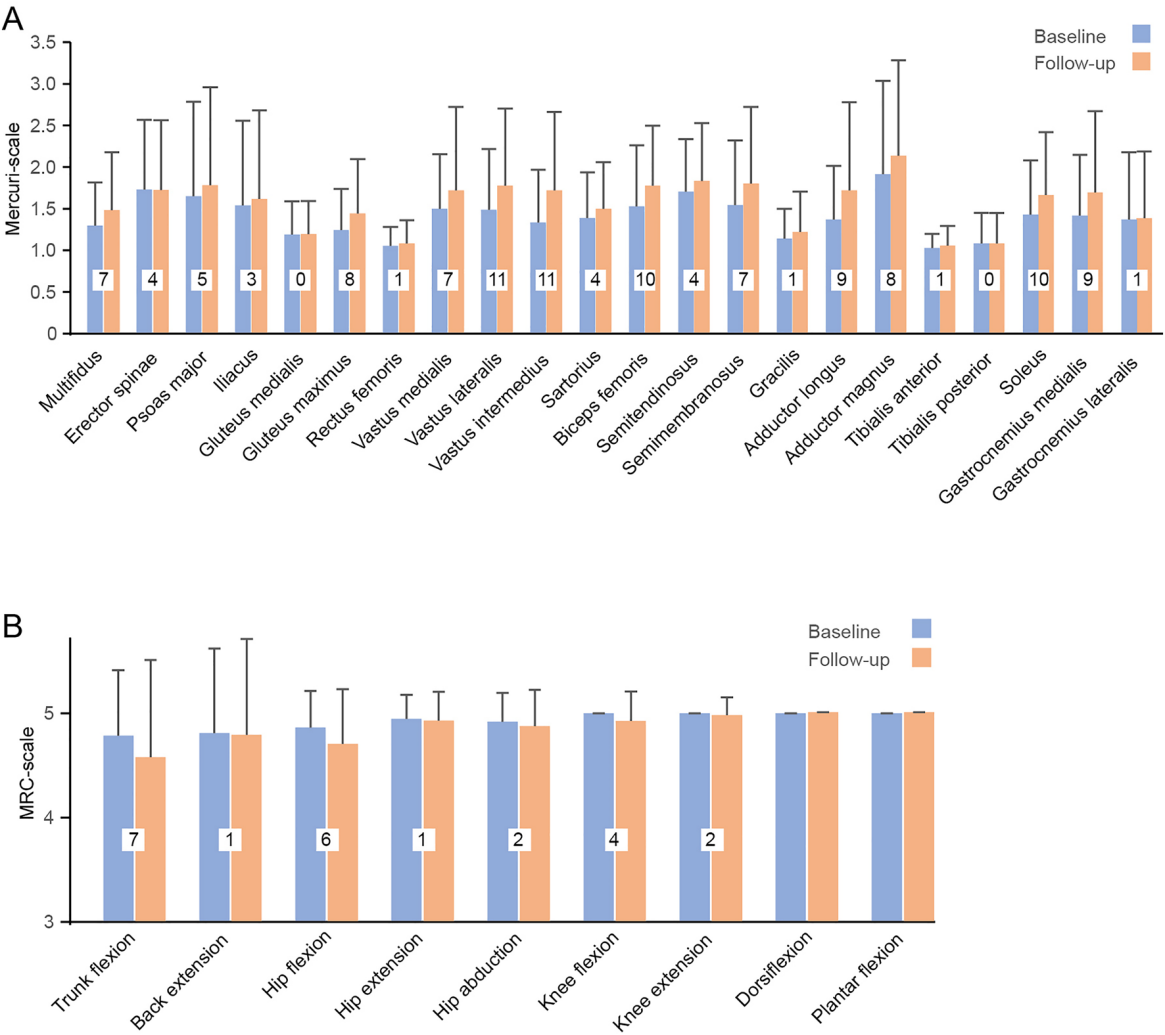
Muscle MRI and muscle strength. **A** Fat replacement on muscle MRI at baseline and follow-up. Twenty-seven participants with a mutation in *CACNA1S* had an increase in fat replacement of muscle during follow-up. The degree of fat replacement was quantified by the Mercuri score. Each muscle was staged from 1 (1 = normal) to 4 (4 = severe fat replacement) Columns represent mean and bars standard deviations. Text boxes includes number of participants with an increase in fat replacement of the specific muscle. **B** Muscle strength at baseline and follow-up. Eleven participants with a mutation in the calcium channel gene *CACNA1S* had a decline in muscle strength during follow-up. Muscle strength was assessed by the Research Council (MRC) scale for manual muscle strength testing ranging from 0 to 5 (5 = normal). Strength of back extension and truncal flexion was assessed as described in Methods. In the figure, we have aligned these measures with the MRC scale. Columns represent mean and bars standard deviations. Text boxes includes number of participants with a decline in muscle strength

Eleven patients with reduced muscle strength at baseline (79% of this group) showed a decline in muscle strength during follow-up (Fig. 1). No participants with normal muscle strength at baseline developed new onset permanent muscle weakness during follow-up. Four of the patients with a decline in muscle strength had no attacks of paralysis during follow-up, and two of these patients had never had attacks of paralysis. The patient heterozygous for the mutation p.R1239H was one of the patients with a decline in muscle strength.

The reduced strength was observed at trunk flexion (*n = *4), back extension (*n = *1), hip flexion (*n = *3), knee flexion (*n = *2), and knee extension (*n = *2) (Fig. 1). The maximum decline on the MRC scale observed in a muscle group was one point. No participants had an increase in muscle strength during follow-up.

### Muscle MRI

**Fig. 2:**
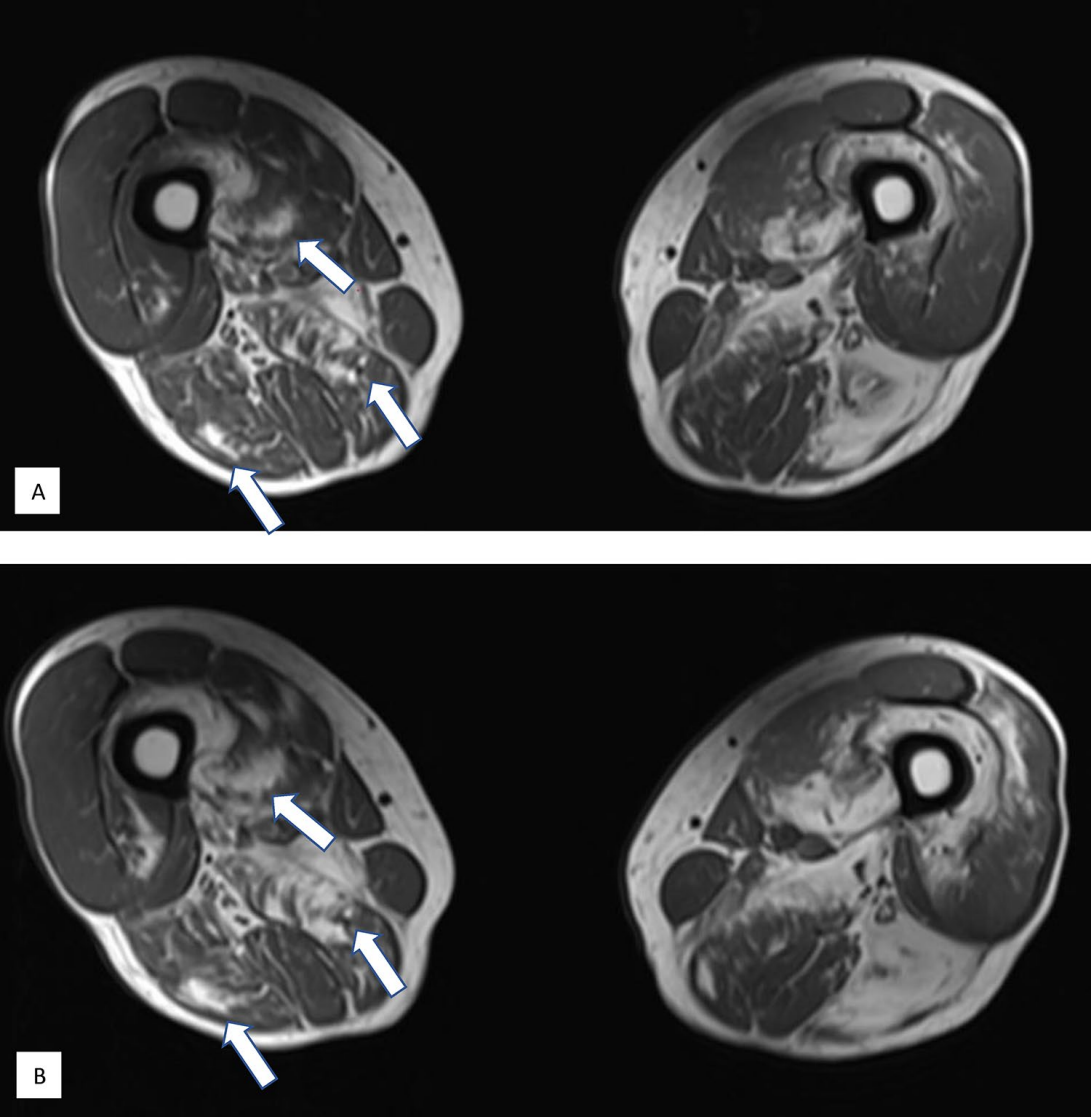
Representative example of progression from baseline in muscle fat replacement of thigh muscles in a 76-year-old (at baseline) male patient with a phenotype of mixed weakness. Axial T1 images at baseline (**A**) and follow-up (**B**). MRI reveals fat replacement, especially of the vastus intermedius, vastus medialis, and the posterior compartments of the thighs. Fat replacement increases from baseline to follow-up (arrows show examples). The patient did not have any attacks of paralysis during the follow-up period

Fat replacement of muscles had increased in 27 patients at follow-up (Fig. 1). Eight of the patients with increased fat replacement had no attacks of paralysis during follow-up, and two of these patients had never had attacks of paralysis. The patient heterozygous for the mutation p.R1239H was one of the patients with an increased fat replacement of muscle. One example of progression on muscle MRI is shown in Fig. 2.

The fat replacement was increased in multifidus (*n = *6), erector spinae (*n = *4), psoas major (*n = *4), Iliacus (*n = *1), gluteus maximus (*n = *8), rectus femoris (*n = *1), vastus medialis (*n = *7), vastus lateralis (*n = *11), vastus intermedius (*n = *13), sartorius (*n = *3), biceps femoris (*n = *9), semitendinosus (*n = *4), semimembranosus (*n = *6), adductor longus (*n = *8), adductor magnus (*n = *6), soleus (*n = *9), gastrocnemius medialis (*n = *9) and gastrocnemius lateralis (*n = *1). The maximum increase in the Mercuri score in the analyzed muscles was one point. No participants had a decline in the Mercuri score during follow-up. Only two patients with normal MRI at baseline (22% of participants in with this finding) had new onset fat replacement at follow-up, and they had both had attacks of paralysis during follow-up. Twenty-five participants with an abnormal MRI at baseline (90% of participants in with this finding) had an increase in fat replacement on the Mercuri score at follow-up. The highest Mercury score at baseline in the groups with and without progression are illustrated in Table 1. The participants with an increase in fat replacement were older at baseline than the participants with no signs of progression during follow-up (32 ± 11 vs 47 ± 16 years, *p < *0.05).

## Discussion

In this follow-up study of adults with HypoPP-causing mutations in *CACNA1S,* the main finding is that permanent muscle weakness and fat replacement on muscle MRI can progress in both patients with and without attacks of paralysis. This is in line with our previous cross-sectional study showing an association between age and muscle weakness and fat replacement of muscle and no association with attacks of paralysis [2].

Periodic paralysis of HypoPP can be treated by normalizing potassium levels during attacks by administration of potassium. The frequency of attacks can in some patients be reduced by treatment with the carbonic anhydrase inhibitors acetazolamide and dichlorphenamide Furthermore, nonpharmacologic interventions such as avoiding trigger factors like a carbohydrate-rich diet and vigorous exercise can reduce the frequency of attacks in some patients [9, 15–17]. Currently, it is not known whether treatment with acetazolamide and dichlorphenamide can prevent the development or the progression of permanent muscle weakness. Our data are too limited to conclude on the effect treatment on progression—for example, only three patients in total were treated with acetazolamide. However, acetazolamide has been shown to prevent vacuolar myopathy in the K + -depleted rats (a model of hypoPP) [18], so it could be interesting to investigate the influence of acetazolamide treatment on myopathy in patients with HypoPP in a large prospective study. Previous studies have shown that permanent weakness and fat replacement on muscle MRI can develop in patients without any periodic paralysis [2, 10] and the present study shows that permanent weakness and fat replacement on muscle MRI can progress in patients with a phenotype with attacks of paralysis during periods with no attacks and also can progress in patients with a phenotype with no attacks of paralysis. Further studies are needed to determine the cause of the permanent weakness and fat replacement of muscle and to determine whether attacks of paralysis influence the development or progression of permanent weakness at all. We have previously shown that autophagy is negatively affected in patients with HypoPP and suggest that this dysfunctional autophagy could be a contributing factor [19].

Most cases of HypoPP (about 60%) are caused by pathogenic variants in the calcium channel gene, *CACNA1S*. The most common variants are R528H and R1239H. Approximately 20% have mutations in the *SCN4A* gene while 20% of cases remain genetically undefined [6, 7]. All participants in this study except one were heterozygous for the p.R528H mutation in *CACNA1S*. The patient with the mutation p.R1239H was one of the patients with progression both in muscle weakness and fat replacement on muscle MRI during follow-up. It could be interesting in future studies to further investigate patients with the pathogenic variant p.R1239H and other HypoPP-causing variants and to compare the results to the result of this study.

A limitation of our study is that muscle strength was monitored by manual muscle testing and fat replacement on muscle MRI by the Mercuri Score. Manual muscle testing is not very sensitive and requires quite a large change in strength to capture decline over time and reliability is limited [20, 21]. Therefore, there is a risk that some of the changes in muscle strength were caused by compromised reliability. We aimed to increase the reliability by eliminating interrater variability as muscle strength was tested by the same examiner in all participants both at baseline and follow-up. Still, changes in muscle strength were captured over time in many of the patients and all patients with a decline in muscle strength had increased fat replacement on muscle MRI. The Mercury score does not detect small changes in fat replacement, and small changes can be missed using this score. Fat replacement of muscle is part of normal aging; however, age-related changes are very limited and considered slowly progressive and besides perhaps in the paraspinal muscle unlikely detectable using the Mercuri score [22]. This, combined with the fact that the follow-up period is relatively short, makes us consider the increase in Mercuri score in this study abnormal and not related to normal aging. Assessment of changes in muscle fat fractions by Dixon MRI in other myopathies such as facioscapulohumeral muscular dystrophy [23], spinobulbar muscular atrophy [24], and skeletal muscle sodium channel disorders [25] has been shown to be more sensitive to identifying progression in myopathy than muscle strength testing. Still, a progression of fat replacement was detected in the majority of the patients in our study. We are currently investigating whether quantitative muscle MRI can detect fat replacement in more patients with HypoPP. If so, Dixon MRI could be a relevant biomarker in future clinical trials investigating treatment effects on progressive myopathy in HypoPP. Our study suggests that both patients with and without periodic paralysis should be included in such trials. Another limitation of our study is the variation in length of follow-up. Some participants with a relatively short follow-up would likely have developed more changes if the follow-up was longer, and some participants with a relatively long follow-up would likely have developed lees changes if the follow-up was shorter. Therefore, in this study, it is not possible to estimate the rate of progression of myopathy.

In conclusion, in this 3-year follow-up study, we found that HypoPP-causing variants in *CACNA1S* can cause a progressive myopathy in both patients with and without attacks of paralysis.

## Data Availability

Data not published within the article will be available upon request.
